# *Trypanosoma cruzi* Mitochondrial Peroxiredoxin Promotes Infectivity in Macrophages and Attenuates Nifurtimox Toxicity

**DOI:** 10.3389/fcimb.2022.749476

**Published:** 2022-02-04

**Authors:** Gabriela Specker, Damián Estrada, Rafael Radi, Lucía Piacenza

**Affiliations:** Departamento de Bioquímica and Centro de Investigaciones Biomédicas (CEINBIO), Facultad de Medicina, Universidad de la República, Montevideo, Uruguay

**Keywords:** *T. cruzi*, nifurtimox, mitochondrial peroxiredoxin, macrophage infection, holdase and peroxidase activities

## Abstract

*Trypanosoma cruzi* is the causative agent of Chagas disease which is currently treated by nifurtimox (NFX) and benznidazole (BZ). Nevertheless, the mechanism of action of NFX is not completely established. Herein, we show the protective effects of *T. cruzi* mitochondrial peroxiredoxin (MPX) in macrophage infections and in response to NFX toxicity. After a 3-day treatment of epimastigotes with NFX, MPX content increased (2.5-fold) with respect to control, and interestingly, an MPX-overexpressing strain was more resistant to the drug. The generation of mitochondrial reactive species and the redox status of the low molecular weight thiols of the parasite were not affected by NFX treatment indicating the absence of oxidative stress in this condition. Since MPX was shown to be protective and overexpressed in drug-challenged parasites, non-classical peroxiredoxin activity was studied. We found that recombinant MPX exhibits holdase activity independently of its redox state and that its overexpression was also observed in temperature-challenged parasites. Moreover, increased holdase activity (2-fold) together with an augmented protease activity (proteasome-related) and an enhancement in ubiquitinylated proteins was found in NFX-treated parasites. These results suggest a protective role of MPX holdase activity toward NFX toxicity. *Trypanosoma cruzi* has a complex life cycle, part of which involves the invasion of mammalian cells, where parasite replication inside the host occurs. In the early stages of the infection, macrophages recognize and engulf *T. cruzi* with the generation of reactive oxygen and nitrogen species toward the internalized parasite. Parasites overexpressing MPX produced higher macrophage infection yield compared with wild-type parasites. The relevance of peroxidase vs. holdase activity of MPX during macrophage infections was assessed using conoidin A (CA), a covalent, cell-permeable inhibitor of peroxiredoxin peroxidase activity. Covalent adducts of MPX were detected in CA-treated parasites, which proves its action *in vivo*. The pretreatment of parasites with CA led to a reduced infection index in macrophages revealing that the peroxidase activity of peroxiredoxin is crucial during this infection process. Our results confirm the importance of peroxidase activity during macrophage infection and provide insights for the relevance of MPX holdase activity in NFX resistance.

## Introduction

*Trypanosoma cruzi* (*T. cruzi*) is the intracellular parasite that causes Chagas disease, which is considered a neglected disease and remains a public health problem in Latin America, estimated to affect approximately 6 million people and with 70 million at risk of infection (Pérez-Molina and Molina, 2018). *Trypanosoma cruzi* has a complex life cycle that involves two hosts, the insect vector and the vertebrate host, and four stages of the parasite. The replicative, non-infective epimastigote stage is found in the insect midgut and, during its migration to the hindgut, differentiates to the infective, non-replicative metacyclic trypomastigotes that can invade different vertebrate cells. Inside cells, trypomastigote transforms into the infective, replicative intracellular amastigote. Following several rounds of replication, the infective blood trypomastigote escapes and disseminates the infection to other tissues. During the life cycle, parasite survival depends on its rapid and efficient adaptation to the distinct environments faced, which is accomplished by extensive biochemical and morphological changes. *Trypanosoma cruzi* is a heterogeneous species, with large genetic variability and different strains that circulate between the vertebrate host and the insect vector. This heterogeneity may explain the variation in the clinical manifestation of Chagas disease and the geographical differences in morbidity and mortality (Laurent et al., 1997; Da Silva Manoel-Caetano and Silva, 2007; McCall and McKerrow, 2014).

Activation of professional phagocytes (macrophages and neutrophils) with the concomitant generation of oxidant species is a medullar innate immune process for the control of acute *T. cruzi* infection. Recent data reinforce the hypothesis that parasites more prepared to deal with the host-oxidative assault are more efficient for the establishment of the infection (Piacenza et al., 2009a; Alvarez et al., 2011; Martinez et al., 2014; Estrada et al., 2018; Piacenza et al., 2019). Nevertheless, *T. cruzi* contains a complex system of antioxidant enzymes and related proteins that allows it to overcome the oxidants, produced by the host immune response (Wilkinson et al., 2000; Mateo et al., 2008; Piacenza et al., 2009b; Wilkinson et al., 2002a; Wilkinson et al., 2002b; Wilkinson et al., 2002c). This antioxidant system has multiple components distributed in all parasite subcellular compartments, where they maintain the redox balance playing a key role in parasite virulence and persistence in tissues (Piacenza et al., 2013; Estrada et al., 2018; Piacenza et al., 2019).

Peroxide metabolism in *T. cruzi* relies on five peroxidases located in different subcellular compartments: heme-containing cytochrome c ascorbate peroxidase, two glutathione-like peroxidases and two peroxiredoxins, ubiquitous peroxidases that depend on cysteine residues to reduce peroxides (Wilkinson et al., 2000; Wilkinson et al., 2002a; Wilkinson et al., 2002b; Wilkinson et al., 2002c; Hugo et al., 2017). Both the cytosolic (CPX) and the mitochondrial peroxiredoxin (MPX) are two cysteine-typical peroxiredoxins which use tryparedoxin (TXN) as reducing substrate. The oxidized TXN is then reduced by trypanothione [T(SH)_2_], a low molecular weight thiol unique to trypanosomatids, at the expense of NADPH. *Trypanosoma cruzi* peroxiredoxins are able to detoxify H_2_O_2_, peroxynitrite (ONOOH), and short-chain organic hydroperoxides (ROOH), and its level of expression has been correlated with the virulence of different strains (Wilkinson et al., 2000; Trujillo et al., 2004; Piñeyro et al., 2008; Piacenza et al., 2009b). In most two cys-typical peroxiredoxins, such as MPX and CPX, the basic functional unit is the dimer, but generally, they are able to establish higher-order structures specifically as octameric, decameric, and dodecameric toroids (Cao and Lindsay, 2017). In recent years, it has been described in many organisms that peroxiredoxins, besides its function as peroxidases, have other activities such as holdase and as part of signaling pathways (Rhee et al., 2012; Perkins et al., 2015; Sobotta et al., 2015). Holdases are part of the molecular chaperone family which prevents aggregation of proteins in an ATP-independent manner (Folgueira and Requena, 2007; Kim et al., 2013). Holdase activity is crucial for cellular protection during different stress conditions that induces protein damage and unfolding with the possible formation of protein aggregates. The interplay between the peroxidase and holdase function of peroxiredoxins is regulated by several environments in the cell such as pH, temperature, ions, posttranslational modifications, and redox balance (Jang et al., 2006; Saccoccia et al., 2012; Morais et al., 2017a). Moreover, holdase activity seems to be related to the oligomeric state of the enzyme, taking into consideration that holdase activity is mainly reported in decameric or higher molecular weight oligomers, which means that conditions that favor the said structure will stimulate this activity in peroxiredoxins. It is proposed that oligomerization dynamics and, therefore, holdase function are dependent on the redox state of the cell, given that oxidized forms of peroxiredoxins, which have mainly dimeric conformations, do not exhibit holdase activity, whereas their reduced or overoxidized forms, which form decamers and/or higher molecular weight oligomers, display holdase activity (Wood et al., 2002; Wood et al., 2003; Rhee and Woo, 2011; Perkins et al., 2015). However, there are reports that also show that oxidized peroxiredoxins are able to acquire high molecular weight conformations, and thus, the precise relationship between redox state and holdase activity will depend on the specific peroxiredoxin under study (Yewdall et al., 2016). Regarding these novel functions, it was found that *T. cruzi* CPX has the ability to prevent aggregation of malate dehydrogenase *in vitro* independently of its redox state (Piñeyro et al., 2019). Furthermore, the crystal structure of the CPX is available and shows that it is a toroid-shaped decamer in its active and reduced form (Piñeyro et al., 2005), like most peroxiredoxins that act as holdases. It was shown that this peroxiredoxin does not disassemble into dimers upon oxidation. Nonetheless, it has not been yet proven *in vivo* that *T. cruzi* peroxiredoxins have holdase activity. In *Leishmania infantum*, another trypanosomatid with long-lasting residence in the macrophage phagosome, it has been reported that mitochondrial peroxiredoxin (*Li*Prx1m) presents holdase activity *in vitro* and that a deficiency in this enzyme yields parasites more sensitive to a rise in temperature (Castro et al., 2011). These deficient parasites also presented a decreased survival in macrophages, but interestingly, they could be rescued by the expression of the *Li*Prx1m in which the peroxidatic cysteine (Cp) was absent, indicating that other activity than the classical peroxidase (i.e., holdase) is involved in protecting amastigotes to macrophage-derived stressors (Castro et al., 2011; Teixeira et al., 2015).

Treatment of Chagas disease relies mainly on two old and poorly specific chemotherapeutic agents: benznidazole (BZ) and nifurtimox (NFX). These drugs have several disadvantages; they are only effective in the acute phase of the disease, treatment may last for months with important side effects, and there are resistant strains (Rassi et al., 2010). Both of these drugs are nitroheterocycle compounds: NFX is a nitrofurane derivative, while BZ is a nitroimidazole. The mode of action of NFX is still elusive and two major mechanisms have been proposed, which agree in the fact that NFX has to be metabolized to act as an anti-trypanosomatid drug. First, NFX toxicity was explained by NFX redox cycling with superoxide radical (O_2_^•−^) and H_2_O_2_ production (Docampo and Stoppani, 1979; Docampo and Moreno, 1984), with a concomitant increase in oxidative stress within the parasite. The drug was thought to be activated by one electron reduction catalyzed by type II nitroreductase (NTR-II), producing nitrofurane radical, that in aerobic conditions gives rise to a futile cycle that results in regeneration of the nitro group and production of O_2_^•−^ which dismutate to H_2_O_2_ and O_2_ catalyzed by superoxide dismutases (SODs) in a diffusion-controlled reaction (McCord and Fridovich, 1969). This postulation is based on the observation that after NFX treatment, parasite extracts showed an increased oxygen consumption and production of O_2_^•−^ as well as in the fact that *Trypanosoma brucei* strains deficient in cytosolic iron SOD (TbSODB1) showed more sensitivity toward NFX (Roberto Docampo and Stoppani, 1979; Prathalingham Radhika et al., 2007). Moreover, it was reported that treatment with NFX leads to depletion of the principal low molecular weight thiols of the parasite, although it was discussed that this could be explained by redox cycling of the drug or by conjugation of thiols with drug metabolites (Maya et al., 1997).

The second mechanism relies on the activation of NFX by a parasite NADH-dependent mitochondrial type I nitroreductase (NTR-I), insensitive to O_2_, that has FMN as cofactor and catalyzes sequential 2-electron reduction steps to yield unsaturated open chain nitriles that have trypanocidal activities with no significant generation of oxidative stress (Boiani et al., 2010; Hall et al., 2011). *Trypanosoma cruzi-*resistant strains have only one copy of *Tc*NTR-I gene, and genetic modification of parasites to give knockout or overexpressers resulted in resistant or hypersensitive parasites to NFX, respectively. This shows a connection between NTR-I activity and nifurtimox toxicity (Wilkinson et al., 2008; Cerecetto and González, 2011).

Taking into consideration the abovementioned results related to the peroxiredoxins of trypanosomatids and its different functions within the cell, we evaluated the ability of *T. cruzi* mitochondrial peroxiredoxin to act as holdase and studied the relative weight of both activities (peroxidase and holdase) toward macrophage and NFX-derived toxicity. The results presented herein contribute to the understanding of the role of *T. cruzi* mitochondrial peroxiredoxin in response to different stress, gaining insights into the redox and holdase function of this peroxidase.

## Results

### Parasites Overexpressing MPX Are More Resistant to NFX Toxicity

**Figure 1:**
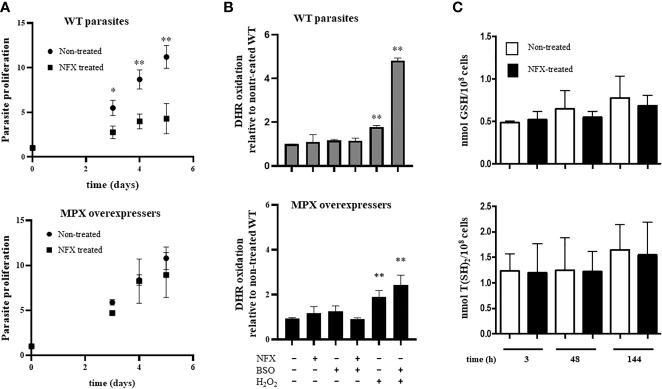
The protective role of mitochondrial peroxiredoxin (MPX) toward nifurtimox (NFX) in epimastigotes without modifying redox state. **(A)** Growth curve of epimastigotes wild type (WT) (upper panel) and MPX-overexpressing parasites (lower panel) in the absence or presence of NFX (7 μM). Parasites were cultured in BHI medium at 28°C and cell density was evaluated every day by absorbance at 600 nm. * denotes statistical differences (*p* < 0.05) and ** statistical differences (*p* < 0.01) compared with non-treated (two-tailed unpaired *t*-test). **(B)** Dihydrorhodamine (DHR) oxidation. Non-treated or overnight BSO-treated parasites were preloaded with DHR (50 μM) and exposed either to NFX (7 μM, 2 h) or H_2_O_2_ (300 μM, 30 min). DHR oxidation was measured in a fluorescence plate reader (Varioskan Flash, Thermo Scientific) at *λ*_ex_ = 485 nm and *λ*_em_ = 520 nm. Upper panel: DHR oxidation in WT parasites. Lower panel: DHR oxidation in parasites overexpressing MPX. Results are expressed as fluorescence units with respect to non-treated WT condition. ** denotes statistical differences (*p* < 0.01) compared with non-treated condition (two-tailed unpaired *t*-test). **(C)** The content of reduced low molecular weight (LMW) thiols in WT epimastigotes (1 × 10^8^ cells) incubated for different times with NFX (7 μM) was determined by HPLC as described. Upper panel: Quantification of GSH in non-treated (white bars) and NFX-treated WT parasites (black bars) at different times. Lower panel: Quantification of T(SH)_2_ in non-treated (white bars) and NFX-treated WT parasites (black bars). Results are expressed as nmol thiol/10^8^ parasites.

**Figure 2:**
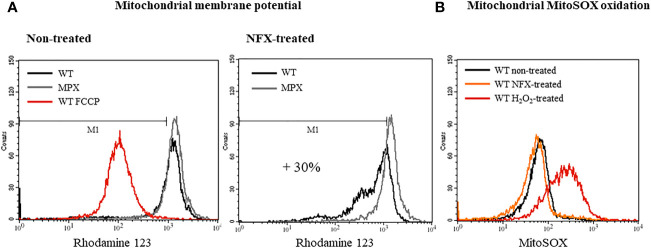
Overexpression of MPX prevents loss of mitochondrial membrane potential in epimastigotes treated with NFX. **(A)** Evaluation of the mitochondrial membrane potential of epimastigotes WT and MPX overexpressers without treatment (left histogram) or treated with NFX (7 μM, 2 h, right histogram) performed with the mitochondrial probe RH123 by flow cytometry. FCCP was used as a control of mitochondrial depolarization (left panel). **(B)** Parasite mitochondrial O_2_^•−^/H_2_O_2_ generation was evaluated by MitoSOX oxidation and analyzed by flow cytometry. Following NFX treatment (7 μM, 2 h) or not (non-treated), epimastigotes were washed with PBS and loaded with MitoSOX (5 μM, 30 min), washed with PBS, and analyzed. The positive control was achieved by treating parasites with H_2_O_2_ (300 µM, 10 min), then washed and loaded with the probe.

In order to evaluate the influence of MPX content in parasites on NFX toxicity, the growth curves of epimastigotes in the absence or presence of the drug in a concentration close to its IC_50_ (7 μM) were performed (Boiani et al., 2010). WT and genetically transformed parasites to overexpress MPX (**Supplementary Figure S1**) (Piacenza et al., 2008) showed a similar growth profile in control conditions (**Figure 1** and **Supplementary Figure S2**), whereas in the presence of NFX, MPX-overexpressing parasites showed an increased resistance to the toxic effects of the drug compared with WT parasites (**Figure 1A**, upper and lower panels). Since MPX is able to detoxify different peroxides and taking into account that this species may be generated during the early stages of NFX metabolism in some circumstances, we searched for the generation of reactive oxygen species following dihydrorhodamine (DHR) oxidation. Parasites treated for 2 h with the drug were loaded with DHR and the formation of RH123, the oxidized product, was evaluated. No increase in probe oxidation was observed in our experimental conditions (**Figure 1B**). Parasite treatment with H_2_O_2_ (300 µM) for 30 min was previously shown to increase intramitochondrial superoxide radical generation (Estrada et al., 2018) and was used herein as a positive control. Increased DHR oxidation was observed in H_2_O_2_-treated parasites, and this was significantly enhanced when parasites were previously (24 h) incubated in the presence of buthionine-S,R-sulfoximine (BSO), an inhibitor of GSH synthesis (Griffith and Meister, 1979; Meister, 1995). In this condition, overexpression of MPX leads to a significantly lower DHR oxidation with respect to WT parasites (**Figure 1B**). The levels of non-protein thiols [i.e., GSH and T(SH)_2_] were evaluated daily during the growth curve in non-treated and NFX-treated parasites by mBrBm derivatization and HPLC-fluorescent detection (**Figure 1C**). GSH (0.6 ± 0.2 nmol/10^8^ cells) and T(SH)_2_ (1.6 ± 0.6 nmol/10^8^ cells) remain unchanged during the 5-day incubation with NFX with intracellular levels in the order of that previously reported (Thomson et al., 2003). Overall, our results suggest that in our experimental conditions, NFX is not generating a redox cycle with the production of reactive oxygen species, and thus, the protective effects observed in MPX-overexpressing parasites seem not to be ascribable to its peroxidase activity. Moreover, since it was proposed that NFX is activated at the parasite mitochondria, we examined the mitochondrial membrane potential after a 3-day parasite culture with the drug by rhodamine-123 incorporation and the site-specific generation of O_2_^•−^/H_2_O_2_ by MitoSOX oxidation (**Figure 2**). NFX treatment of WT parasites caused a significant decrease (30%) of mitochondrial membrane potential with respect to non-treated parasites, whereas no changes in mitochondrial membrane potential were observed for MPX-overexpressing parasites (**Figure 2A**). FCCP, a mitochondrial uncoupler, was used as a positive control of mitochondrial depolarization. Finally, MitoSOX oxidation was evaluated in parasites following a 2-h incubation with NFX, and also, non-detectable oxidation of the probe was observed in drug-treated parasites. Parasite treatment with H_2_O_2_ as described above (300 µM, 30 min) was used as a positive control (**Figure 2B**), given that this treatment leads to mitochondrial superoxide radical generation (Estrada et al., 2018). The results presented herein suggest that enhanced content of MPX protects parasites for the NFX toxicity at the mitochondrial compartment.

### NFX Induces Enhanced MPX Protein Expression in WT Parasites

**Figure 3:**
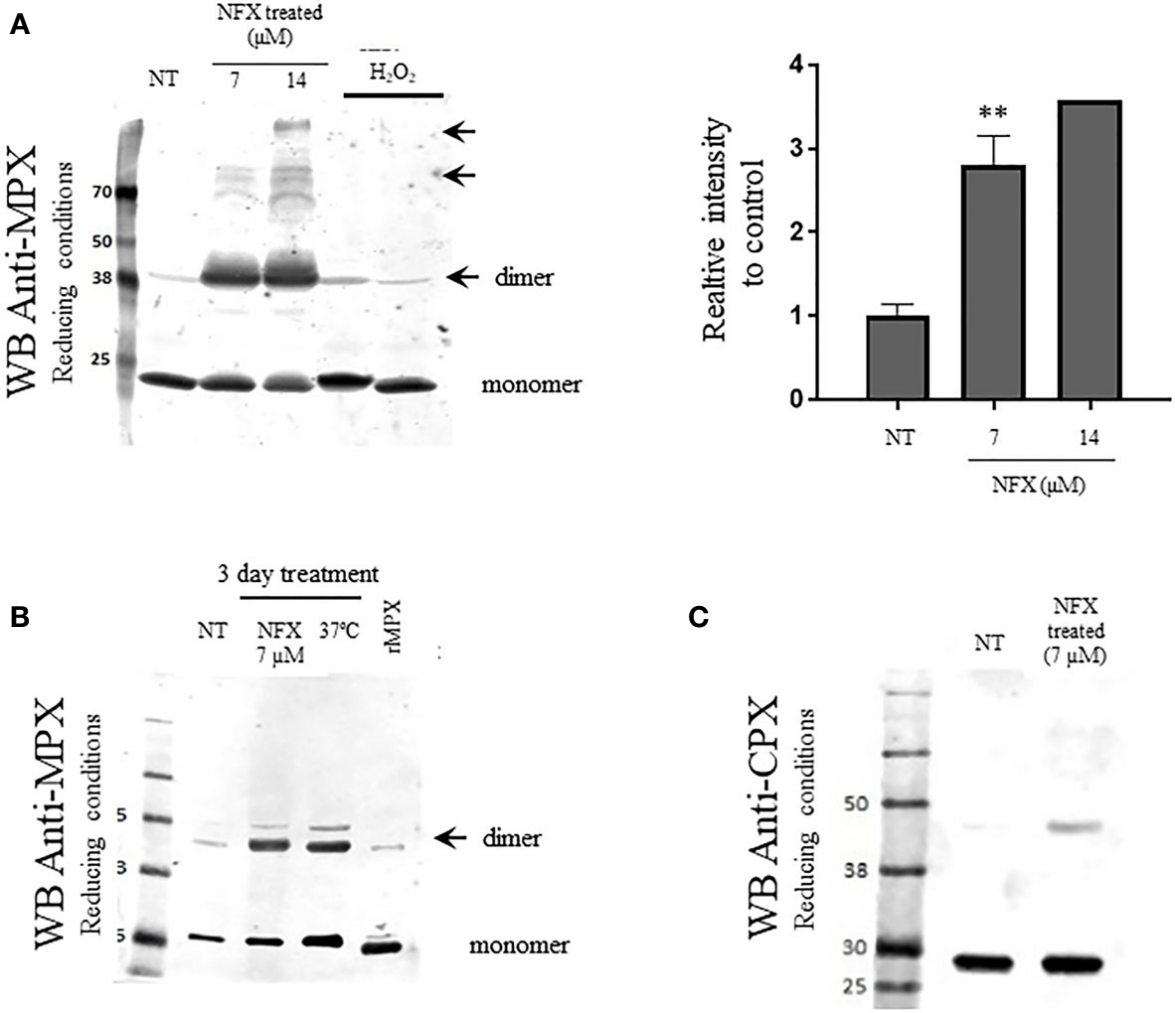
Increased expression of MPX but not cytosolic peroxiredoxin (CPX) in WT parasites exposed to NFX. **(A)** Epimastigotes (WT) were grown in the absence or presence of NFX (7–14** **µM, 3 days). After treatment, parasites (1 × 10^8^ cells) were collected, washed, and resuspended in PBS buffer containing NEM (10 mM). Total protein extract (50 μg) was run on SDS-PAGE (12%) under reducing conditions (DTT, 1 mM) and Western blot for MPX and CPX was performed as described. Parasites incubated with H_2_O_2_ (300 µM, 30 min) was performed as control. Right panel: Relative quantification of band intensity with respect to control condition. Results are means ± SEM of relative densitometric quantitation by LI-COR Image Studio of at least three independent determinations; ** denotes statistical differences (*p* < 0.01). For quantification, Coomassie blue staining of the different samples (50 μg) was used as loading control (**Supplementary Figure S3**). NT, non-treated. NFX: treated 3 days with NFX 7 or 14 μM. H_2_O_2_: treated with H_2_O_2_ 300 μM for 10 min. **(B)** Evaluation of mitochondrial MPX content in parasites grown for 3 days in the presence of NFX [as in panel **(A)**] or at 37°C. Western blot anti-MPX of protein extract was performed as in panel **(A)**. Recombinant MPX (rMPX, 5 µg) was run as control. NT, non-treated. NFX: treated 3 days with NFX 7 μM. 37°C: grown 3 days at 37°C. MPXr, recombinant MPX. **(C)** Western blot anti-CPX of protein extract of control parasites or grown in the presence of NFX, performed as in panel **(A)**. NT, non-treated. NFX: treated 3 days with NFX 7 μM.

The content of MPX in WT parasites was evaluated following a 3-day treatment with NFX (7 and 14 μM). As shown in **Figures 3A, B**, MPX expression was significantly increased in NFX-treated parasites in a dose-dependent manner (2.5- and 3.5-fold increase) strengthening the hypothesis of a protective role of this enzyme toward the damage exerted by NFX. No changes in protein content were observed in the cytosolic peroxiredoxin (CPX) and other *T. cruzi* peroxiredoxin with holdase activity *in vitro* (**Figure 3C**) (Piñeyro et al., 2019) in agreement with the mitochondrial activation of NFX. Interestingly, NFX treatment leads to the generation of non-reducible (with freshly prepared DTT or phosphine) dimers and higher aggregates that were not observed with H_2_O_2_ treatment (**Figures 3A, B**, arrows**)**. Moreover, an increase in MPX content and the same non-reducible MPX dimers were observed after a temperature challenge by growing parasites at 37°C for 3 days (**Figure 3B**, arrows). This shift in temperature occurs during the life cycle of *T. cruzi* when the insect-derived metacyclic trypomastigotes (28°C) invade the vertebrate host (37°C). Furthermore, in order to evaluate if the increase in MPX content is also induced by other anti-*T. cruzi* drugs (BZ, azasterol, ketoconazole), parasites were incubated for 3 days with the IC_50_ of each drug. Only BZ treatment enhanced the MPX content (**Supplementary Figure S4**) in agreement with its mitochondrial-dependent NTR-I activation. BZ toxicity has been proposed to occur *via* reduced intermediates that covalently modified low molecular weight thiols and different macromolecules with no generation of reactive oxygen species (Trochine et al., 2014). The above results ruled out the generation of oxidative stress after NFX treatment, and we aimed to evaluate the protective role of the MPX holdase activity.

### *Trypanosoma cruzi* Mitochondrial Peroxiredoxin Exhibits Holdase Activity

**Figure 4:**
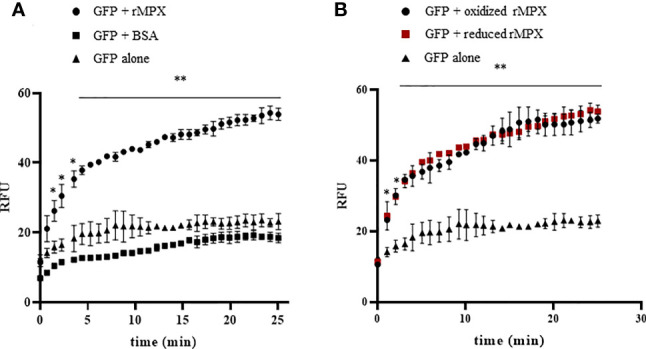
Holdase activity of recombinant MPX. **(A)** Holdase activity of recombinant MPX (rMPX) was evaluated with the GFP refolding assay as described in the *Materials and Methods* section. GFP was incubated in refolding buffer alone or in the presence of recombinant MPX (rMPX, 50 μM) or BSA (50 μM). * denotes statistical differences (*p* < 0.05) and ** statistical differences (*p* < 0.01) compared with GFP alone (two-tailed unpaired *t*-test). **(B)** Holdase activity of recombinant MPX (rMPX, 50 μM) in its reduced (DTT, 1mM) and oxidized state. * Denotes statistical differences (*p* < 0.05) and ** statistical differences (*p* < 0.01) compared with GFP alone (two-tailed unpaired *t*-test). There were no statistical differences between reduced and oxidized MPX.

To assess whether MPX is able to function as holdase, preventing protein aggregation, it was first tested if recombinant enzyme could enhance the refolding of GFP. This fluorescent protein loses its structure after a short incubation at low pH and, therefore, its fluorescence properties. GFP can refold and regain its fluorescent structure at a slow rate in the absence of holdases; however, when the refolding buffer is supplemented with a protein that can act as a holdase, the process of refolding is accelerated (Mares et al., 2011). As shown in **Figure 4A**, the presence of recombinant MPX (rMPX, 50 μM) causes GFP (0.5 µM) to recover its fluorescence more efficiently. As a control condition, incubation of unfolded GFP with an unrelated protein that lacks holdase activity [bovine serum albumin (BSA), 50 μM) did not accelerate GFP refolding (**Figure 4A**). In case of MPX, as described also for CPX (Piñeyro et al., 2019), holdase activity is not dependent on its redox state since both reduced and oxidized rMPX and the mutant lacking the peroxidatic cysteine (**Supplementary Figure S5**) exhibit holdase activity (**Figure 4B**).

**Figure 5:**
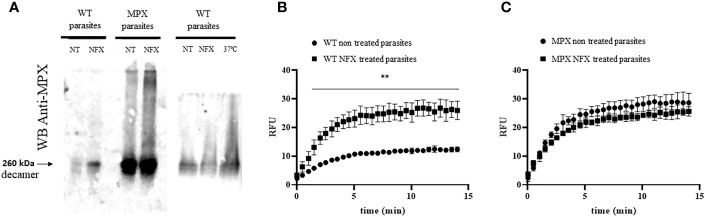
Holdase activity of parasite MPX. **(A)** Mitochondrial peroxiredoxin ability to acquire high molecular weight aggregates in parasite was studied by native gel electrophoresis (8%) and Western blot anti-MPX of total protein extract (obtained as in **Figure 3**, 50 μg) of WT or MPX-overexpressing parasites grown in the absence (non-treated, NT) or presence of NFX (NFX-treated, 7 μM, 3 days; left panel) or at 37°C (grown 3 days at 37°C; right panel). NT, non-treated. NFX: treated 3 days with NFX 7 μM. 37°C: grown 3 days at 37°C. **(B, C)** High molecular weight protein extract (>100 kDa) was obtained from epimastigotes (1 × 10^9^) non-treated or treated with NFX (7 μM, 3 days) as described in the *Materials and Methods* section. Level of holdase activity in high molecular weight fraction (300 μg) was assessed by GFP refolding assay. **(B)** WT parasite extracts, non-treated and treated with NFX. **(C)** MPX-overexpressing parasite extracts non-treated and treated with NFX. ** denotes statistical differences (*p* < 0.01) compared with WT non-treated parasite extracts (two-tailed unpaired *t*-test). There were no statistical differences between extracts from MPX parasites non-treated and treated with NFX.

**Figure 6:**
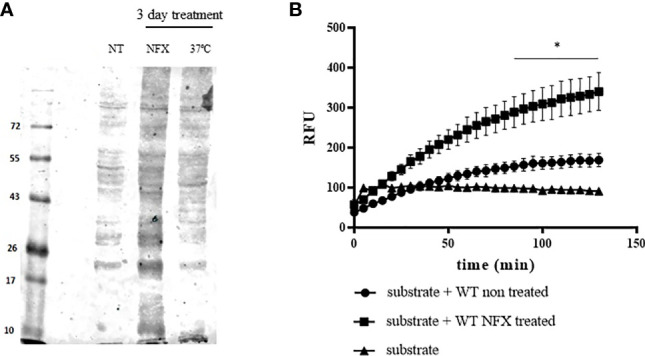
Increased protein ubiquitination and mitochondrial protease activity in WT parasites treated with NFX. **(A)** Evaluation of proteins conjugated to ubiquitin in WT parasites non-treated (NT) or treated with NFX (7 μM, 3 days) or grown at 37°C (3 days). Following treatment, parasites were collected by centrifugation and washed in PBS, and the protein extract was obtained as described. Total protein extract (50 μg) was run on SDS-PAGE (12%) and Western blot anti-ubiquitin (Abcam/ab7780) was performed. **(B)** Protein extracts derived from parasites (1 × 10^8^) grown in the absence or presence of NFX were tested for proteosome-related protease activity. Total protein extracts (300 μg) were incubated at 28°C in buffer Tris–HCl pH 7.4 containing 50 μM of the fluorogenic substrate Suc-Leu-Leu-Val-Tyr-AMC (50 μM). AMC release was followed fluorometrically at *λ*_ex_ = 365 nm and *λ*_em_ = 440 nm. * denotes statistical differences (*p* < 0.05) compared with WT non-treated parasite extracts (two-tailed unpaired *t*-test).

Holdase activity of some peroxiredoxins may rely on the gaining of higher structure levels with the formation of high molecular weight aggregates (Wood et al., 2003; Jang et al., 2004). In this sense, we evaluated whether parasite MPX could acquire high molecular weight aggregates after NFX incubation (7 µM, 3 days). For this purpose, the protein extracts from non-treated and NFX-treated parasites were separated on native 8% polyacrylamide gels and subjected to Western blot with anti-MPX antibody. As shown in **Figure 5A**, NFX and/or temperature treatment (37°C, 3 days) leads to the formation of high molecular weight aggregates of MPX that were not observed in non-treated parasites. Then, we enriched the parasites extracts (NFX-treated or not) in high molecular protein or protein aggregates (>100 kDa) and used these enriched extracts to search for MPX holdase activity in order to establish if NFX treatment could lead MPX to gain this function. As seen in **Figure 5B**, this high molecular weight extract obtained from WT parasites treated with NFX displayed an enhancement in holdase activity with respect to non-treated parasites. In the MPX-overexpressing parasites, no difference in holdase activity was observed between non-treated and NFX-treated parasite extracts (**Figure 5C**). This result indicates that NFX treatment of WT parasites causes MPX to enhance its expression and to gain holdase activity probably due to NFX-induced mitochondrial protein mis-/unfolding and that this function is related to the protective role of MPX toward NFX. In this line, NFX-treated parasites presented enhanced levels of protein ubiquitination (**Figure 6A**) and a significant increase in the mitochondrial proteosome-related protease activity (**Figure 6B**), suggesting that mitochondrial protein damage is being elicited by NFX metabolism.

### Selective Inhibition of MPX Peroxidase But Not Holdase Activity by Conoidin A

**Figure 7:**
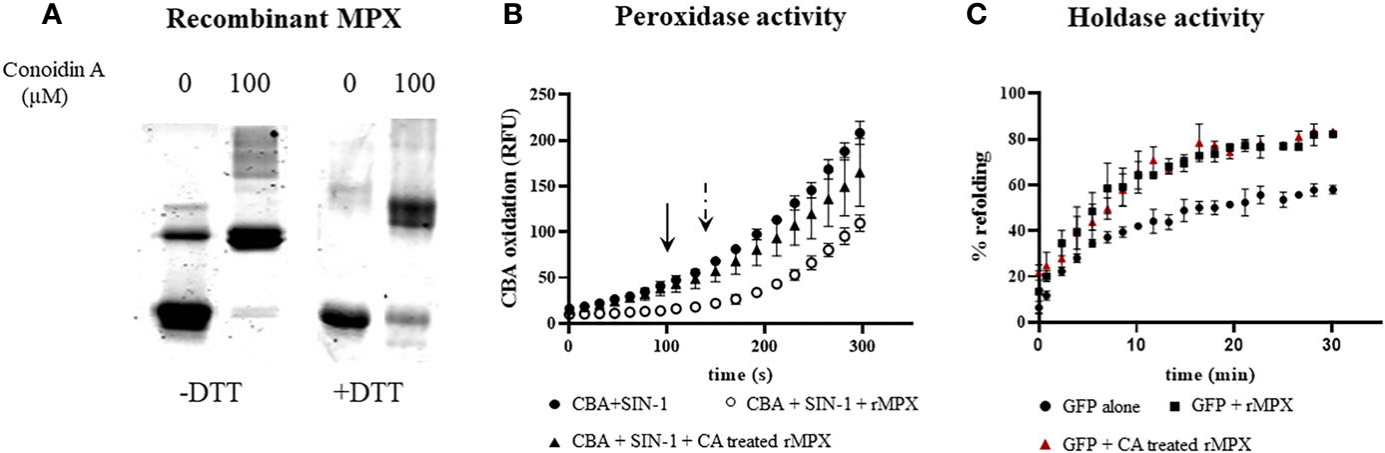
Peroxidase but not holdase activity inhibition by conoidin A on recombinant MPX. **(A)** Recombinant MPX (5 μM) treated with conoidin A as described in the *Materials and Methods* section. Samples were run on SDS-PAGE (12%) under reducing (DTT, 1 mM) or non-reducing conditions. Coomassie blue staining is shown. Conoidin A resulted in the formation of non-reducible adducts of MPX. **(B)** Measurement of peroxidase activity of previously reduced MPX (5 μM) control or incubated with conoidin A by its ability to decompose peroxynitrite produced by SIN-1 (2 mM, 3 μM/min), in the presence of CBA (50 µM). CBA oxidation was followed fluorometrically at *λ*_ex_ = 365 nm and *λ*_em_ = 440 nm. Arrow indicates the time lapse for non-treated MPX peroxynitrite detoxification (i.e., ~100 s), and dotted arrow indicates the time lapse for CA-treated MPX (i.e., ~180 s). **(C)** Measurement of holdase activity by the GFP refolding assay of recombinant MPX (50 μM) (squares) and recombinant MPX pretreated with conoidin A (triangles); control (circles) is the spontaneous refolding of GFP. There were no statistical differences between non-treated MPX and CA-treated MPX.

With the aim to evaluate the role of peroxidase vs. holdase activity of MPX during macrophage infections, we examined the effects of conoidin A (CA), a previously reported cell-permeable covalent inhibitor of the peroxidase activity of some peroxiredoxins, by alkylating or crosslinking the catalytic cysteines (Liu et al., 2010; Brizuela et al., 2014). We first search for the ability of CA (100 µM, 1 h) to produce MPX crosslinking. As shown in **Figure 7A**, Western blot of recombinant MPX treated for 1 h with CA (100 µM) revealed the presence of non-reducible aggregates of MPX indicating that CA reacts and crosslinks recombinant MPX. Then, peroxidase activity of recombinant pre-reduced rMPX (5 µM), control or previously incubated with CA (100 µM), was followed by the inhibition of boronate oxidation (CBA, 50 µM) in the presence of controlled fluxes of peroxynitrite generated by SIN-1 (3.5 µM ONOO^−^/min). We took advantage of the practicality of evaluating peroxidase activity through this fast and simple assay, given that peroxynitrite is a key mediator of parasite control in the context of infection (Alvarez et al., 2011). MPX was able to inhibit ONOOH-dependent CBA oxidation (~100 s, arrow), whereas when MPX was previously incubated with CA, peroxidase activity was inhibited, and CBA oxidation occurred with practically the same characteristics as when MPX was not added to the assay (**Figure 7B**). Finally, holdase activity was evaluated in MPX previously incubated with CA, which showed the same ability to aid in the GFP refolding than that of non-treated reduced MPX. Thus, CA treatment was unable to inhibit holdase activity, confirming that CA inhibits peroxidase but not holdase activity in recombinant MPX.

### MPX Peroxidase Activity Is Important for Parasite Survival in Macrophage Infections

**Figure 8:**
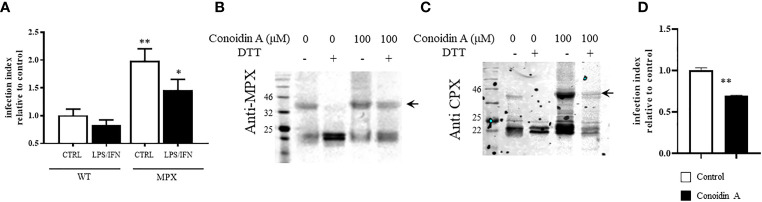
MPX peroxidase activity is required for trypomastigotes to thrive in macrophage infections. **(A)** Naive or immunostimulated macrophages were infected with culture-derived trypomastigotes (WT or MPX overexpressers) at a parasite:cell ratio of 3:1. Following 2 h, non-internalized parasites were removed and infection proceeded for 24 h. Intracellular amastigotes were counted fluorometrically (DAPI staining) with at least 1,000 macrophages/condition recorded. Results are expressed as the infection index of at least three experiments calculated expressing the number of amastigotes/100 macrophages relative to WT infection. ** and * denote statistical differences (*p* < 0.01 and *p* < 0.05, respectively) with respect to WT control condition. **(B, C)** Western blot anti-MPX and anti-CPX of protein extracts of culture-derived trypomastigotes treated with conoidin A (100 μM, 1 h) in DMEM medium at 37°C. Total protein extracts were obtained as described and samples containing 50 μg protein were analyzed by SDS-PAGE (12%) under reducing (DTT, 1 mM) or non-reducing conditions. **(D)** Conoidin-treated trypomastigotes [the same conditions as panel **(B)**] were used to infect naive macrophages as described in panel **(A)**, and intracellular amastigotes were counted following 24 h of infection. Results are expressed as the infection index calculated expressing the number of amastigotes/100 macrophages relative to WT control. ** denotes statistical differences (*p* < 0.01).

In order to clearly state the role of MPX peroxidase activity during macrophage invasion, we performed infection experiments in naive and immunostimulated macrophages for peroxynitrite generation. As shown in **Figure 8A**, overexpression of MPX rises by 2-fold the ability of parasites to establish the infection in both naive and activated macrophages. This result indicates the crucial role of MPX for parasite survival. In order to discriminate the relative weight of peroxidase vs. holdase activity during the macrophage infection process, we conducted experiments using trypomastigotes preincubated with CA. First, we evaluated the ability of this inhibitor to react with parasite MPX. For this, epimastigotes were exposed to CA (100 µM, 1 h) and protein extracts subjected to Western blot anti-MPX and anti-CPX under reducing and non-reducing conditions. As shown in **Figures 8B**, **C**, non-reducible MPX and CPX adducts were formed in the presence of CA (arrows) as previously described for other peroxiredoxins (Nguyen et al., 2013). This peroxiredoxin CA-induced adducts were stable for at least 4 h; adducts were no longer observed 24 h later indicating the ability of the parasite to degrade them. Then, CA-treated parasites were used to infect naive macrophages; since the parasite resides in the macrophage phagosome for at least 2 h, peroxiredoxin adducts are still present during parasite internalization in the macrophage phagosome. Finally, CA treatment leads to a significant decrease in the number of intracellular amastigotes following 24 h of infection (**Figure 8D**). This means that peroxidase activity of peroxiredoxins is crucial for parasite survival during macrophage infection, reinforcing the importance of the peroxidase activity of peroxiredoxins in parasite resistance and evasion of the macrophage-derived oxidative assault.

## Discussion

The importance of enzymes of the antioxidant network for parasite protection toward exogenous or endogenously derived reactive oxygen and nitrogen species has been previously documented (Piacenza et al., 2007; Piacenza et al., 2013), and in particular, the role of peroxiredoxins in the protection of the parasite against macrophage-derived oxidants has been established (Piacenza et al., 2008; Alvarez et al., 2011; Piñeyro et al., 2011). However, little is known regarding the possibility that, as in other organisms, *T. cruzi* peroxiredoxins have other roles in dealing with different kinds of stress. Herein, we showed the involvement of MPX in protecting parasites from NFX toxicity. The observations that increased levels of MPX help parasites to overcome the toxicity of NFX (**Figures 1A** and **2A**) and that incubation in these conditions causes overexpression of MPX in wild-type parasites (**Figure 3A**) but not that of the cytosolic CPX (**Figure 3C**) clearly indicate that MPX is involved in the parasite protective response toward the drug. Overexpression of MPX was also observed in BZ-treated parasites (**Supplementary Figure S4**), which may be explained by the fact that BZ metabolism relies on the same mitochondrial nitro reductase NTR-I (as for NFX activation), which means that the mitochondria is the first organelle affected by the action of these drugs (although each drug may have different targets). However, other drugs like ketoconazole and azasterol (McCabe et al., 1984; Goad et al., 1989; Urbina et al., 1995; de Souza and Rodrigues, 2009) that inhibit *T. cruzi* growth by affecting sterol synthesis did not affect the MPX content in parasites (**Supplementary Figure S4**). This result points to the conclusion that MPX overexpression is a specific protective response that occurs when the mitochondria is faced with stress.

Since different hypotheses were built in order to explain NFX toxicity, we first assessed the possibility that the ability of MPX to detoxify peroxides was the basis for the observed protection. Nonetheless, we did not observe any evidence of a significant increase in reactive oxygen species production and/or mitochondrial superoxide generation in wild-type parasites treated with NFX (**Figures 1B**, **2B**), so we could not conclude that peroxidase activity explains the protection conferred by MPX toward NFX in this experimental condition. These results are in agreement with previous reports that showed that NFX and BZ are activated by mitochondrial NTR-I in a sequential 2-electron reduction steps that does not involve the generation of reactive oxygen species (Wilkinson et al., 2008; Cerecetto and González, 2011).

The protective role of *L. infantum* mitochondrial peroxiredoxin (*Li*mTXNPx) during thermal stress and also during macrophage infections was previously proposed. This protective role was fulfilled by the expression of a peroxidase inactive version of the enzyme (Castro et al., 2011). Taking this result into consideration and knowing the ability of *T. cruzi* CPX to prevent protein aggregation *in vitro* (Piñeyro et al., 2019), we consider the possibility that the protection conferred by MPX toward NFX may involve the alternative functions of peroxiredoxins, such as the yet unexplored holdase activity.

In this sense, we evaluated the ability of recombinant MPX to aid in GFP refolding and established that *in vitro* this enzyme increases the rate by which unfolded GFP regains its native structure (**Figure 4A**). Knowing that holdase activity of several peroxiredoxins is proposed to be modulated by the redox state of the protein, we evaluated MPX holdase activity in its reduced and oxidized state as well as for the catalytic mutant (**Supplementary Figure S5**). Similar holdase activity was observed in both redox states of the enzyme (**Figure 4B**), in agreement with what was previously shown for *T. cruzi* cytosolic CPX (Piñeyro et al., 2019). The observation that recombinant MPX showed holdase activity (**Figure 4**) prompted us to analyze whether MPX is able *in vivo* to acquire high molecular weight (HMW) oligomers that are related to holdase activity in other peroxiredoxins, including *T. cruzi* CPX (Wood et al., 2002; Piñeyro et al., 2019). We found that MPX can acquire this oligomeric structure in the parasite, and interestingly, HMW aggregates are significantly increased in NFX-treated parasites (**Figure 5A**). These same MPX aggregates were observed in parasites under thermal stress, which suggest a connection between NFX and temperature challenge (**Figure 5A**).

After establishing that MPX has holdase activity *in vitro* and that in parasites it can acquire HMW oligomers that increase its abundance in response to NFX, we aimed to study if this is also accompanied by an increase in holdase activity. For this purpose, we measured holdase activity in the HMW protein extracts (>100 kDa) of parasites not treated and treated with NFX. For WT parasites in which an increase in MPX content was observed following NFX treatment, a significant increase in holdase activity with respect to untreated parasites was observed (**Figure 5B**). This result first establishes MPX holdase activity *in vivo* and, second, could indicate that parasites increase and activate anti-aggregation systems in response to NFX, suggesting that the drug mitochondrial metabolism may generate a rise in damaged and/or unfolded proteins. Importantly, the MPX-overexpressing parasites with enhanced MPX content did not show an increase in holdase activity after NFX treatment suggesting that they can deal with the NFX unfolded protein damage response without the need to further increase MPX expression (**Figure 5C**).We have yet to establish the oligomerization state for MPX and the minimum level of MPX that allows the oligomerization needed to function as a holdase, considering that peroxiredoxins of trypanosomatids seem to have holdase activity in its decameric form (Teixeira et al., 2015; Piñeyro et al., 2019).

To evaluate if NFX causes the unfolding or misfolding of proteins tagging them for degradation, we search for protein ubiquitination and increase in protease activity after NFX treatment of WT parasites. We found an increase in protein ubiquitination and enhancement in proteosome-related protease activity following NFX treatment (**Figures 6A, B** **)**, which may link NFX toxicity with the increase of MPX expression in WT parasites and the need to gain holdase activity to counteract the toxic effects. The unfolding of proteins could be a consequence of the production of reactive nitriles generated by NFX metabolism by parasite mitochondrial NTR-I, an enzyme that is not found in the vertebrate host (Wilkinson et al., 2008; Cerecetto and González, 2011). This hypothesis is in agreement with the observation that MPX expression increases in parasites grown under thermal stress as previously reported (Pérez-Morales et al., 2012) and as also observed in this work (**Figures 3B**, **5A**). *Trypanosoma cruzi* life cycle entails multiple changes in environmental conditions such as temperature, pH, and oxidative stress. Parasites have to adapt to these varying environments in order to survive and be able to establish the infection. Besides the antioxidant enzyme system described by our group and others (Piacenza et al., 2008; Piñeyro et al., 2008; Piacenza et al., 2009b; Martínez et al., 2019), the parasite has evolved other mechanism to deal with stress such as molecular chaperones, which highlights the importance of maintaining protein homeostasis (Shonhai et al., 2011). The observations reported herein could indicate that NFX alters protein homeostasis in *T. cruzi* causing damage to the mitochondria and that the increase in MPX holdase activity could be part of the response to protein unfolding. The fact that thermal stress, known to require a chaperone response (Sanchez and Lindquist, 1990; Hübel et al., 1997; Jäättelä, 1999), induces MPX overexpression as previously reported (Pérez-Morales et al., 2012) and shown herein supports this hypothesis. Further work is required to characterize the alteration in proteostasis caused by NFX and if this is linked to the toxicity produced by the drug.

Taking into consideration the MPX holdase function described herein, it was considered whether this activity may be of relevance in the context of macrophage infection in which reactive oxygen and nitrogen species are being generated (Alvarez et al., 2011).

In an attempt to disclose the relevance of both MPX activities (peroxidase vs. holdase) and knowing that *T. cruzi* knockout parasites are extremely difficult to obtain, we evaluate the ability of CA as a possible inhibitor of peroxidase but not holdase activity, since it reacts with the peroxidatic cysteine that is not necessary for holdase activity (Teixeira et al., 2015; Piñeyro et al., 2019). First, we assessed if recombinant MPX reacts with CA which would generate the reported adducts inhibiting peroxidase but not holdase activity. MPX-CA treatment generates the non-reducible protein adducts and inhibits peroxidase activity, but this treatment does not affect MPX holdase activity (**Figure 7**).

Previous studies from our group have shown the ability of MPX to cope with exogenous or endogenously produced H_2_O_2_ and peroxynitrite (Piacenza et al., 2008), but until now, the potential protection conferred by MPX in macrophage infections is not studied. First, it was proven that MPX contents are important for parasite survival on both naive (H_2_O_2_ production) and immune-stimulated macrophages for peroxynitrite generation (**Figure 8A**). This result is in agreement with MPX peroxidase activity, since inside the phagosome the parasite is exposed to peroxynitrite and H_2_O_2_ that can diffuse inside the internalized parasite reaching the parasite mitochondria (Piacenza et al., 2008; Alvarez et al., 2011), or alternatively, it can be generated inside the parasite by means of the diffusion of its precursors nitric oxide and superoxide radicals (Piacenza et al., 2009a; Martínez et al., 2019). Then, in order to assess the possibility to inhibit MPX peroxidase activity in the parasite, and whether this inhibition may influence *T. cruzi* ability to establish the infection, we first evaluated if CA generates MPX adducts *in vivo.* As shown in **Figure 8B**, CA also generates non-reducible covalent adducts of MPX *in vivo* similar to the effect observed in the recombinant protein (**Figure 7A**), indicating that CA is able to act *in vivo.* Unfortunately, CA acts in both *T. cruzi* peroxiredoxins, and thus, the unique role of MPX in macrophage infections is not disclosed (**Figure 8C**). Nevertheless, the role of the peroxidase and/or holdase activities of both peroxiredoxins can be addressed using this inhibitor. We found that CA-treated parasites have a diminished infection index (**Figure 8D**), showing the importance of peroxidase activity to overcome macrophage-derived oxidants in divergence to what was previously observed for *Lm* mitochondrial peroxiredoxin (Castro et al., 2011). This result does not discard the fact that MPX holdase activity could be relevant in the infection process. Ongoing experiments are focused on addressing the relevance of MPX in the context of NFX treatment during macrophage infections.

With the observation that MPX is overexpressed in wild-type parasites when exposed to NFX and BZ (**Figure 3A** and **Supplementary Figure S4**) and knowing that the mitochondrial metabolism of these drugs gives rise to different toxic and reactive intermediates (Cerecetto and González, 2011; Hall and Wilkinson, 2012; Trochine et al., 2014), we propose that the increase in MPX expression observed may be caused by an enhancement in the modified/damaged macromolecules at the mitochondrial compartment. To further understand if MPX is a direct target for metabolites derived from NFX or if its overexpression is induced by other effects caused by the drug, further experiments need to be performed including the characterization of the adducts observed in **Figure 3A**. It was previously shown that there is a distinct MPX content among natural occurring strains and that it has a positive correlation with parasite virulence (Piacenza et al., 2009b). Taking into account that MPX overexpression protects parasites from NFX toxic effect (**Figure 1**), we proposed that the MPX content may be one of the factors that contributes to the differences in NFX sensitivity reported for different *T. cruzi* strains (Murta et al., 1998).

In conclusion, our results demonstrate that MPX is able to function not only as an efficient peroxidase but also as a holdase, preventing protein aggregation. We observed that peroxidase function is crucial for macrophage infection, but MPX holdase activity may be relevant in other conditions, such as thermal or drug-related stress. We have yet to unravel how these two functions are modulated and which conditions could favor holdase activity. There are many factors proposed to impact on holdase activity of peroxiredoxins, such as redox state, ion concentration, and thermal activation (Wood et al., 2002; Morais et al., 2017; Teixeira et al., 2019). Our results point to the fact that holdase activity of MPX is not related to its redox state. However, given that we observed overexpression and high molecular weight aggregates after thermal stress, it would be interesting to deepen our understanding of the effect of temperature on MPX function during the infection process.

## Materials and Methods

### Parasite Culture

*Trypanosoma cruzi* epimastigotes from the CL-Brener (wild type, WT) were cultured at 28°C in brain–heart infusion (BHI) medium as described previously (Piacenza et al., 2001). Parasites overexpressing MPX were obtained as previously described and cultured in BHI containing geneticin (G418, 200 μg/ml). Briefly, the complete gene sequences of the enzyme were cloned into the trypanosomal vector pTEX-9E10 (Invitrogen, Massachusetts, United States) and a ligation was performed to insert a c-Myc-derived epitope (9E10) in-frame at the 3′ end of the genes to produce the construct pTEX-MPX-9E10 (Wilkinson et al., 2000; Piacenza et al., 2008). Peroxidase activity of MPX was reported to be ∼1.9 higher in overexpressing cells (Wilkinson et al., 2000). The increase in MPX expression as well as mitochondrial localization of the protein in the transfected parasites (∼4 times) is shown in the **Supplementary Material** (**Figure S1**).

### Growth Curves With Nifurtimox

Epimastigotes (1 × 10^6^ cells/ml starting culture condition) were grown in BHI medium at 28°C for 5 days in the absence or presence of NFX (7 μM, Sigma). The density of the cultures was evaluated by absorbance at 600 nm every 24 h (Cary UV-Vis spectrophotometer, Agilent). In order to compare different growth curves, we used the parasite proliferation index in which absorbance at 600 nm is expressed relative to the *A*_600 nm_ at the first day of the experiments, and the graphs obtained with this index are plotted as mean ± SD of three independent determinations.

### Mitochondrial Membrane Potential

Rhodamine 123 (RH123, Invitrogen), which accumulates in the mitochondria in a potential-dependent manner, was used as a flow cytometer probe to assess mitochondrial membrane potential. Non-treated parasites (1 × 10^7^ cells) or parasites treated with NFX were washed in PBS and loaded with 5 μg/ml RH123 for 15 min at 28°C, washed in PBS, and analyzed by a flow cytometer (FACS-Calibur, Becton Dickinson). FCCP (trifluoromethoxy carbonylcyanide phenylhydrazone, 1 μM, Sigma) was used as a positive control for mitochondrial depolarization. In order to compare different conditions, the M1 region of low RH123 fluorescence (associated with mitochondrial depolarization) was defined.

### Dihydrorhodamine Oxidation and BSO Treatment

After the different treatments, parasites (1 × 10^7^ cells) were collected by centrifugation and incubated for 30 min at 28°C in dPBS containing DHR (50 μM, Molecular Probes). Then, cells were washed in dPBS in order to eliminate non-incorporated DHR. Detection of intracellular RH123, the oxidation product of DHR, was performed after exposure to the different experimental conditions using black 96-well plates and a fluorescence plate reader at 28°C (Varioskan Flash, Thermo Scientifics) at *λ*_ex_ = 485 nm and *λ*_em_ = 520 nm. Overnight incubation with BSO 500 µM, an inhibitor of glutathione synthesis, was used to decrease parasite low molecular thiols which reduces parasite ability to detoxify H_2_O_2_ (Thomson et al., 2003).

### Low Molecular Weight Thiol Quantification

Parasite GSH and T(SH)_2_ levels were quantified in epimastigotes (WT and MPX overexpressers) incubated or not with NFX every day, for 5 days. Parasites were collected and washed in PBS and resuspended at a cell density of 1 × 10^9^ cells/ml in 100 μl HEPES buffer (40 mM, pH 8.0) containing monobromobimane (0.2 mM). Following incubation at 70°C for 3 min, protein precipitation was performed with methanesulfonic acid (4 M, 100 μl) for 2 h on ice and centrifugation at 13,000*g* for 30 min was also performed. Non-protein thiols were separated by reverse phase ion-pairing HPLC on an Agilent C18 column and analyzed through fluorometric detection as previously described (Krauth-Siegel et al., 1995). Quantitation and validation were accomplished using T(SH)_2_ (kindly provided by Luise Krauth-Siegel) and GSH standards (Sigma) and results expressed as nmol/10^8^ parasites.

### Parasite Extracts and Western Blotting

Epimastigotes treated or not with NFX (3–5 days) were collected at 2,000*g* for 10 min at 25°C, washed, and resuspended in PBS. Lysis was accomplished by freeze and thaw cycles in liquid nitrogen, the extract was centrifuged at 12,000*g* for 30 min at 4°C, and the supernatant was collected. For the enrichment in the parasite high molecular weight aggregates, cell extracts of 1 × 10^9^ parasites were filtered using an Amicon^®^ Ultra-0.5 ml Centrifugal Filter Unit with 100 kDa cutoff.

Parasite extracts were run on polyacrylamide gels (native, 8% or SDS, 12%) under reducing (DTT, 1 mM) or non-reducing conditions using loading buffer without or with SDS [30 mM Tris–HCl, pH 6.6, 1% (w/v) SDS, and 5% (v/v) glycerol]. Loading buffer was added to the supernatant and samples were stored at −20°C until used. Samples were electroblotted into nitrocellulose membranes and equal protein loading was confirmed by Ponceau or Coomassie blue staining. Membranes were blocked in non-fat dry milk (5% in PBS, 1 h) and probed with anti-*Tc*MPX and anti-*Tc*CPX antibodies [kindly provided by Dr. Carlos Robello (Piñeyro et al., 2008)] and anti-ubiquitin antibody (Abcam/ab7780). Reactive proteins were visualized by incubating with secondary antibodies IRE-Dye 800 and/or 680 (LI-COR Biosciences) diluted 1:15,000 (LI-COR Biosciences) and visualized using an infra-red analyzer (Odyssey, LI-COR Biosciences). Band intensity was measured using the program Li-COR Image Studio.

### Purification of Recombinant Proteins

*Escherichia coli* (BL21 strain) transfected with plasmid containing either His-tagged, MPX, MPX-C81S (mutation of the peroxidatic cysteine), or roGFP (kindly provided by Dr. Shane Wilkinson, Dra. Dolores Piñeyro, and Dr. Marcelo Comini, respectively) both with ampicillin resistance was grown in terrific broth to an optical density at 600 nm between 0.6 and 0.8 AU. The expression of the enzymes was induced by addition to the culture of isopropyl-β-D-thiogalactopyranoside (IPTG, 1 mM). Following an overnight incubation at 22°C, the bacteria were collected by centrifugation at 3,000*g*, resuspended in lysis buffer, and sonicated (6 cycles, 1 min each). The lysate was centrifuged 30 min at 12,000*g* and 4°C. The supernatant was collected for purification by affinity chromatography using a nickel column and following supplier instructions (HiTrap Chelating HP, GE Healthcare). Protein elution was monitored by absorbance at 280 nm and the purity of the fractions analyzed by SDS-PAGE with Coomassie blue staining.

### Evaluation of Holdase Activity

Chaperone activity was evaluated as previously described (Mares et al., 2011). Briefly, green fluorescent protein (GFP) is unfolded by incubation with HCl (62.5 mM) in denaturation buffer [Tris–HCl 50 mM pH = 7.5, containing EDTA (0.3 mM) and DTT (1 mM)] for 1 min and transferred to freshly prepared refolding buffer [Tris–HCl 50 mM pH 7.5, containing MgCl_2_ (25 mM), KCl (100 mM), and DTT (0.3 mM)] at a final GFP concentration of 0.5 µM. GFP refolding is monitored fluorometrically (*λ*_ex_ = 485 nm, *λ*_em_ = 538 nm) using a microplate reader (Varioskan Flash, Thermo Scientific). For the evaluation of holdase activity of recombinant MPX (rMPX), the protein was previously reduced (or not, oxidized state) by incubation with DTT (1 mM) for 1 h. DTT was then removed by Micro Bio-Spin™ Size Exclusion Spin Columns (Bio-Rad). Protein was quantified by absorbance at 280 nm, and 50 µM of rMPX was used in the assays. For the assessment of the holdase activity in the high molecular weight protein extracts, these were obtained as described from 1 × 10^9^ parasites, and the protein extract obtained (300 μg) was used in the GFP refolding assay. Bovine serum albumin (BSA, 50 μM) that lacks holdase activity was used as control.

### Assessment of Peroxidase Activity

Peroxidase activity was evaluated indirectly by a competition assay between peroxynitrite-dependent oxidation of coumarin boronic acid (CBA, *k* ~ 1 × 10^6^ M^−1^ s^−1^; pH 7.4 and 25°C) that yields a fluorescent product (coumarin) in the presence and/or absence of rMPX (*k* = 1.8 × 10^7^ M^−1^ s^−1^ at pH 7.4, 25°C) (Piñeyro et al., 2011). Briefly, peroxynitrite released by 3-morpholinosydnonimine hydrochloride (SIN-1, 2 mM) was determined by measuring DHR oxidation (*ϵ*_500_ = 78,800 M^−1^ cm^−1^) as described previously (Radi et al., 2001). Peroxynitrite-dependent (peroxynitrite flux ~ 3 µM/min) oxidation of CBA (50 µM) was followed at *λ*_ex_ = 332 nm and *λ*_em_= 470 nm in the presence or absence of pre-reduced rMPX (5 µM). The decomposition of peroxynitrite by MPX reduces the oxidant generation of coumarin in the first 100 s (lag phase observed in the presence of rMPX with respect to SIN-1 condition) and, thus, CBA oxidation.

### Proteosome-Related Protease Activity

Endopeptidase activity was measured using the fluorogenic substrate Suc-Leu-Leu-Val-Tyr-AMC (CAS 94367-21-2, Santa Cruz), and the hydrolysis of this peptide was followed by the fluorescence of AMC (7-amino-4-methylcoumarin, *λ*_ex_ = 365 nm; *λ*_em_ = 440 nm), which is released upon protease activity. Endopeptidase activity in parasite protein extract was evaluated as follows: parasites (3 × 10^8^ cells) were collected after NFX treatment (3 days, 7 µM), washed in PBS, and lysed by freeze and thaw cycles; the extract was centrifuged at 12,000*g* for 30 min at 4°C; and the supernatant was collected. Endopeptidase activity in the supernatant (150 μg) was measured at 28°C in buffer Tris–HCl pH 7.4 by the increase in fluorescence of AMC in a microplate reader (Varioskan Flash, Thermo Scientific). The initial concentration of fluorogenic substrate was 50 μM.

### Parasite Differentiation (Metacyclogenesis)

Metacyclic trypomastigotes were obtained by chemical differentiation as described previously (Isola et al., 1986; Piacenza et al., 2009b). Briefly, epimastigotes from a 5-day culture were collected by centrifugation at 800*g* for 10 min at 25°C, washed, and resuspended in 10 ml TAU (triatomine artificial urine) [NaCl, 190 mM; KCl, 17 mM; MgCl_2_, 2 mM; CaCl_2_, 2 mM; sodium phosphate buffer, 8 mM (pH 6.0); and sodium bicarbonate, 0.035%] at 3 × 10^8^ cells/ml. After incubation at 28°C for 2 h, the parasites were transferred and diluted in TAU3AAG (TAU three amino acids plus glucose) medium (TAU, pH 6.0) supplemented with L-proline, 10 mM; L-glutamate, 50 mM; L-aspartate, 10 mM; and L-glucose, 10 mM to 3 × 10^6^ cells/ml, following incubation for 96 h at 28°C as described previously (Bonaldo et al., 1988).

### Conoidin A Treatment of Recombinant MPX and Parasites

For the inhibition assays, recombinant MPX was reduced by incubation with DTT 1 mM for 1 h. Then, the reducing agent was removed by Bio-Rad Micro Bio-Spin™ Size Exclusion Spin Columns (Bio-Rad). Reduced rMPX was incubated with CA 100 µM for 1 h, and after incubation, the excess of the inhibitor was removed by Bio-Rad Micro Bio-Spin™ Size Exclusion Spin Columns (Bio-Rad).

To evaluate the inhibition of parasite peroxiredoxin, epimastigotes or trypomastigotes were collected and resuspended in medium (BHI or DMEM) at a density of 1 × 10^8^ cells/ml. CA was added up to a concentration of 100 µM and incubated for further 30 minutes at 28°C (epimastigotes) or 37°C (trypomastigotes). After incubation, parasites were washed with PBS, collected, and used in Western blot or infection assays. The ability of CA to crosslink rMPX was evaluated by SDS-PAGE and Coomassie staining under reducing and non-reducing conditions (freshly prepared DTT and/or phosphine, 1 mM). In parasites, the crosslink was evaluated by SDS-PAGE followed by Western blot anti-MPX under reducing and non-reducing conditions (freshly prepared DTT and/or phosphine, 1 mM).

### Macrophage Culture and Infection

The murine macrophage cell line J774A.1 [American Type Culture Collection (ATCC-TIB-67)] was cultured in DMEM (Sigma) supplemented with L-glutamine (2 mM), penicillin (100 units/ml), streptomycin (100 mg/ml), and 10% heat-inactivated fetal bovine serum at 37°C in a 5% CO_2_ atmosphere.

Metacyclic trypomastigotes obtained as described above were used to infect confluent Vero cells (American Type Culture Collection) at 37°C in a 5% CO_2_ atmosphere. Culture-derived trypomastigotes were used to infect macrophages seeded in Lab-Tek tissue culture chamber with or without stimulation with IFN-γ (100 U/ml) and LPS (5 µg/ml) in a parasite:cell ratio of 3:1 (Alvarez et al., 2011). After 2 h, no engulfed parasites were removed by washing twice in PBS (pH 7.4) and cells were further incubated for 24 h in DMEM at 37°C. The infected cells were fixed in fresh formaldehyde solution [4% (v/v) in PBS] for 10 min at room temperature, washed with PBS, and permeabilized for 5 min with Triton X-100 (0.1%, v/v) in PBS. The number of parasites per macrophage was determined by DAPI staining (5 mg/ml). Preparations were analyzed using a fluorescence microscope (Nikon Eclipse TE-200) at a magnification of ×100, and digital photographs of infected cells were recorded. At least 1,000 cells from three independent experiments were counted. Results are expressed as the number of amastigotes per 100 cells (% of infection) relative to the control and represent the mean of three independent experiments. For some experiments, the culture-derived trypomastigotes were preincubated with CA as described above.

## Data Availability Statement

The original contributions presented in the study are included in the article/**Supplementary Material**. Further inquiries can be directed to the corresponding author.

## Author Contributions

GS: cell culture, infection experiments, enzyme activities, flow cytometry and HPLC experiments, data collection, analysis, interpretation, and writing of the article. DE: flow cytometry and HPLC experiments. RR: data interpretation and critical revision of the article. LP: conception and design of the work, data interpretation, and writing of the article. All authors contributed to the article and approved the submitted version.

## Funding

GS and DE were funded by fellowships from the Comisión Académica de Posgrado, Universidad de la República and Agencia Nacional de Investigación e Innovación, respectively. This work was supported by grants from Universidad de la República: Espacio Interdisciplinario (to RR), Comisión Sectorial de Investigación Científica, I+D-2017 (to LP and GS), I+D-2020 (to LP, DE and GS), and Grupos 2018 (to RR). Additional support was obtained from Programa de Desarrollo de Ciencias Básicas (PEDECIBA, Uruguay) and the Uruguayan National System of Researchers (SNI) from ANII.

## Conflict of Interest

The authors declare that the research was conducted in the absence of any commercial or financial relationships that could be construed as a potential conflict of interest.

## Publisher’s Note

All claims expressed in this article are solely those of the authors and do not necessarily represent those of their affiliated organizations, or those of the publisher, the editors and the reviewers. Any product that may be evaluated in this article, or claim that may be made by its manufacturer, is not guaranteed or endorsed by the publisher.
