# Supplementary material for: Trypanosoma cruzi Mitochondrial Peroxiredoxin Promotes Infectivity in Macrophages and Attenuates Nifurtimox Toxicity
Source: Front Cell Infect Microbiol. 2022 Feb 4;12:749476. doi: 10.3389/fcimb.2022.749476 (PMC8855072; doi:10.3389/fcimb.2022.749476)
Supplement: Supplementary file 1 [file Presentation_1.pdf]

Supplementary Material; Specker et al. Figure 1, panel A

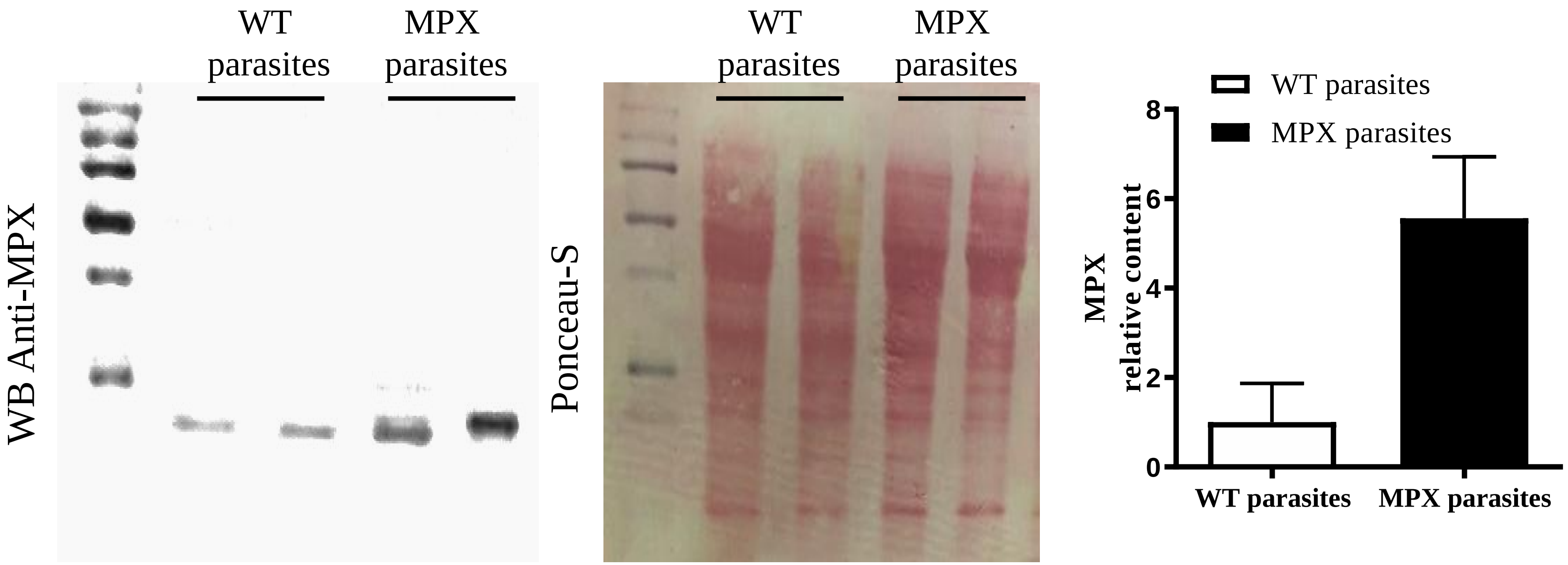

Supplementary Material; Specker et al.
Figure 1, panel B and C

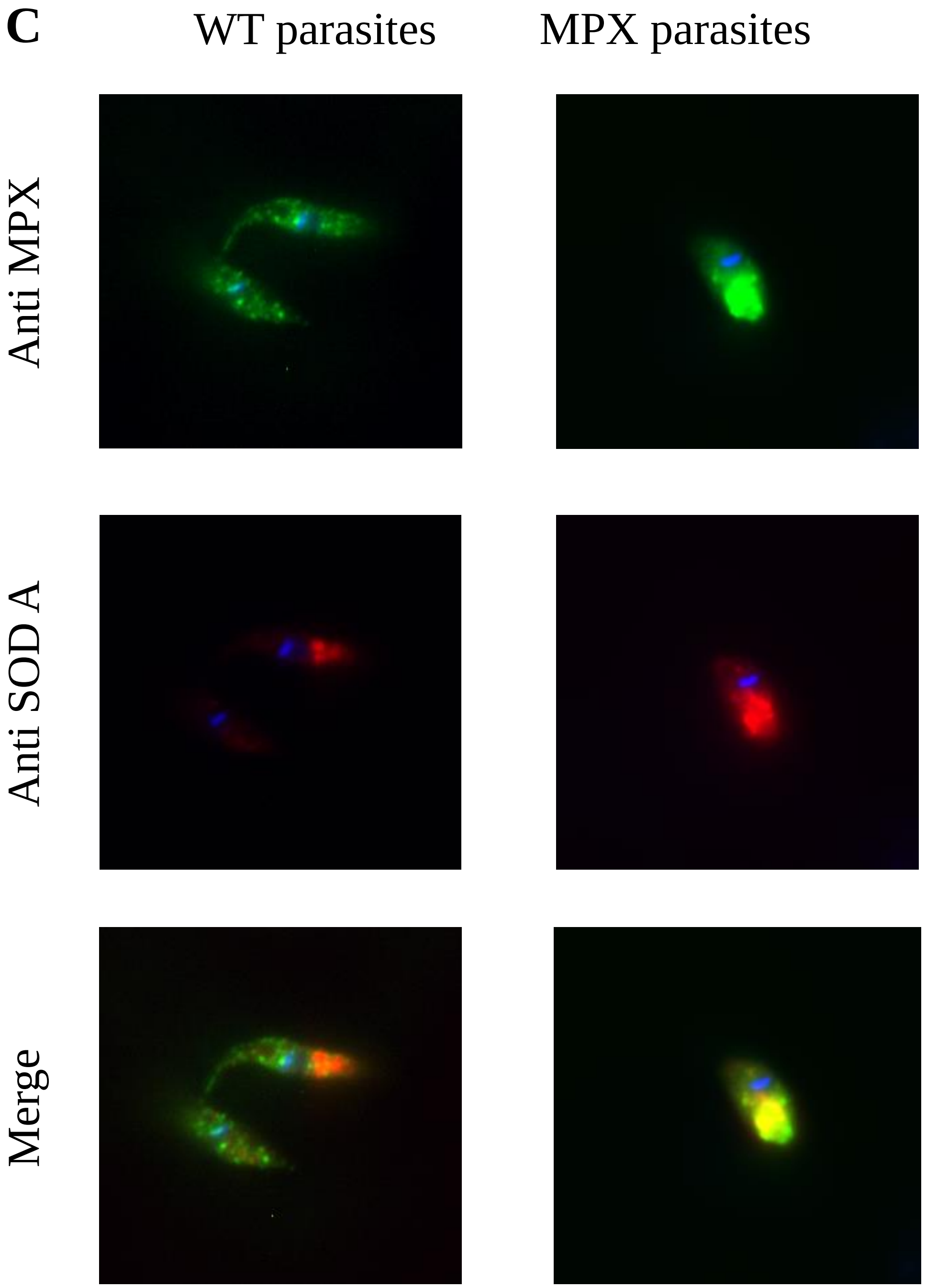

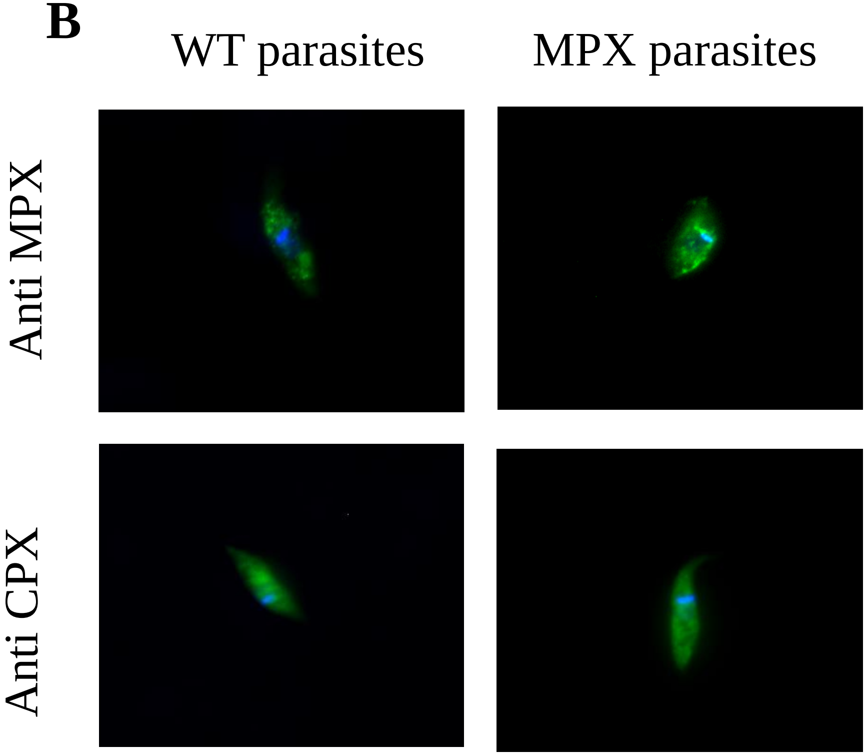

Figure 1S. Expression of MPX in transformed epimastigotes: overexpression and localization

A. Epismatigotes WT and transformed with pTEX_MPX_9E10 as previously described (Piacenza et al., 2008; Wilkinson et al., 2000) were grown in BHI for five days. Protein extract were obtained by 3 freeze and thaw cycles and centrifugation at 12000g for 30 minutes at 4ºC. Total protein extracts (50 μg) were run on SDS-PAGE (12%) under reducing conditions (DTT, 1 mM) and western blot for MPX performed as described. Equal loading was checked by Ponceau-S red staining of the membrane. Lower panel: Relative quantification of band intensity respect to control condition.

B. Immunodetection of MPX and CPX in *T. cruzi* epimastigotes from WT and MPX overexpressers. Anti MPX and CPX is shown in green, FeSOD A in red, and DAPI/DNA in blue. (Magnification: 100×).

C. Immunodetection of MPX and Fe-SODA in *T. cruzi* epimastigotes from WT and MPX overexpressers. Anti MPX and CPX is shown in green, FeSODA in red, and DAPI/DNA in blue. (Magnification: 100×).

Supplementary Material; Specker et al. Figure 2.

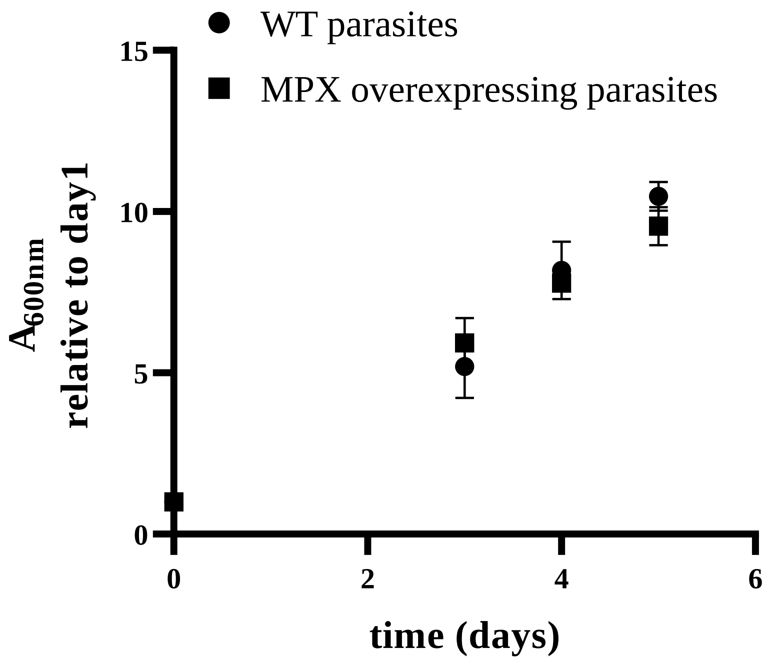

Figure 2S. Growth curve of epismatigotes WT and MPX overexpressing parasites non treted conditions. Parasites were cultured in BHI medium at 28ºC and cell density evaluated every day by absorbance at 600 nm.

Supplementary Material; Specker et al. Figure 3.

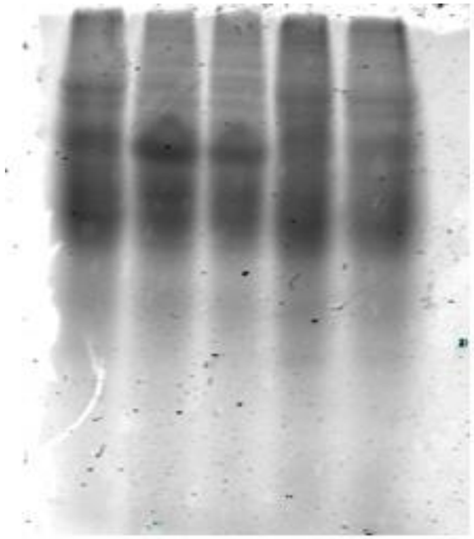

Figure 3S. Loading control of western blots shown in Figure 3 and 5.

A. Protein extracts (50 μg) used for the evaluation of MPX expression in WT in presence of NFX (Figure 3A) and assessment of the ability of MPX to form high molecular weight aggregates (Figure 5A) were run in SDS PAGE (12%) under the same conditions and stained with coomassie blue. Densitometry of coomassie staining was used as loading control.

Supplementary Material; Specker et al. Figure 4.

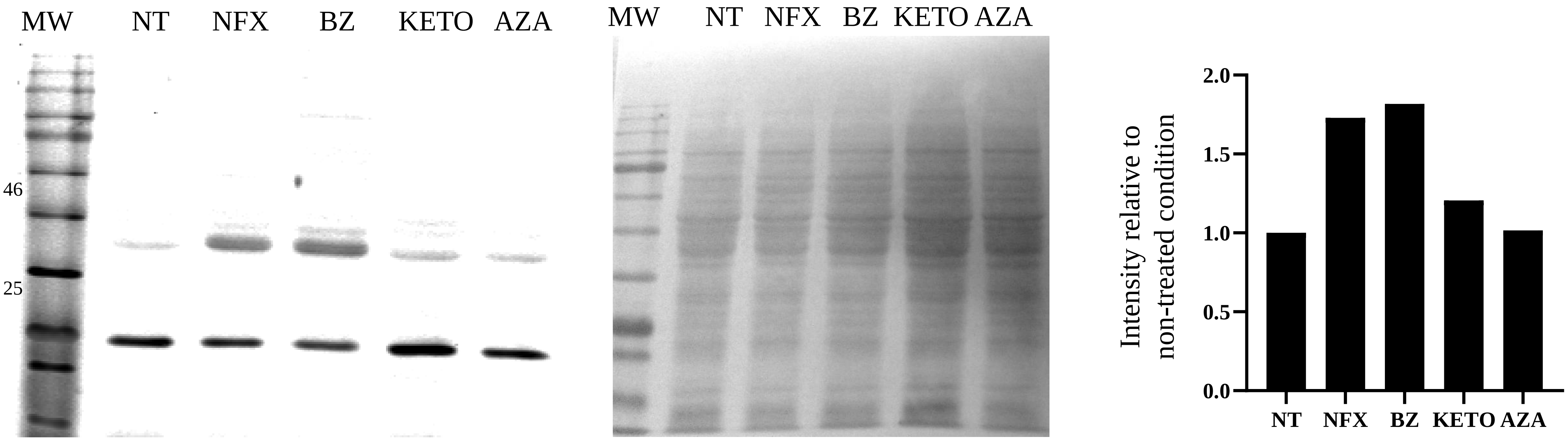

Figure 4S. Expression of MPX in parasites treated with different anti-trypanosomatids

A. WT Epismatigotes grown in BHI for three days in the absence (non-treated) or in presence of the drugs in a concentration similar to its IC_{50}, [NFX]= 7 µM, [BZ]= 14 µM, [KETO]= 2 µM [AZA]= 7 µM. After treatment parasites ($1x10^8$) were collected and protein extract were obtained by 3 freeze and thaw cycles and centrifugation at 12000g for 30 minutes at 4ºC. Total protein extracts (50 µg) were run on SDS-PAGE (12%) under reducing conditions (DTT, 1 mM) and western blot for MPX performed as described. Equal loading was checked by Ponceau-S red staining of the membrane. Right panel: Relative quantification of band intensity respect to control condition normalized by Ponceau-S intensity. NT: Non treated; NFX: Nifurtimox treated; BZ: Benznidazol trated; KETO: Ketoconazol treated; AZA: Azasterol treated

Supplementary Material; Specker et al. Figure 5.

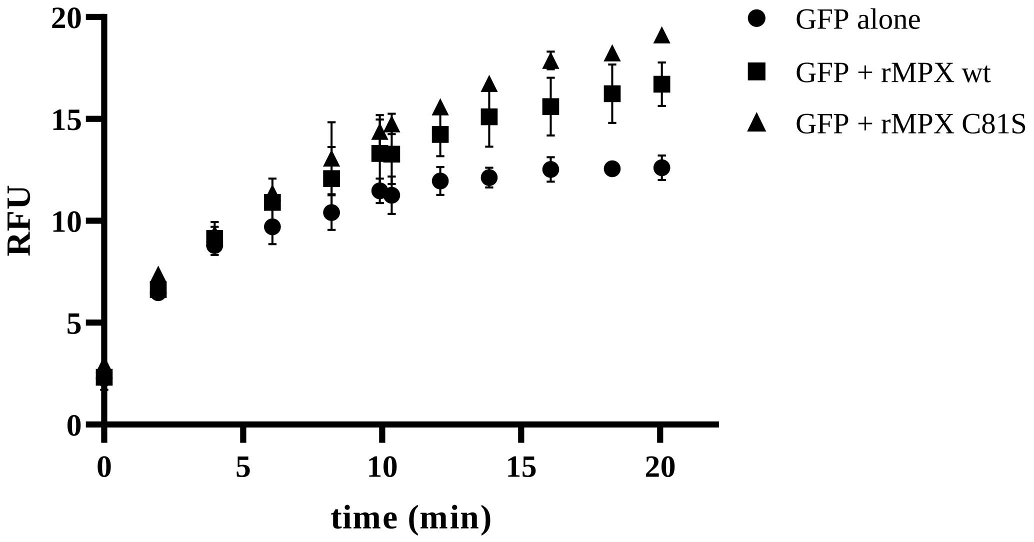

Figure 5S Holdase activity of recombinant MPX (rMPX, 12 μM) wild-type and with its peroxidatic cysteine replaced with serine (rMPX C81S, 12 μM) was evaluated with the GFP refolding assay as described in the material and methods section.
